# The fundamental: Ungrounded or all-grounding?

**DOI:** 10.1007/s11098-019-01332-x

**Published:** 2019-08-14

**Authors:** Stephan Leuenberger

**Affiliations:** Philosophy, School of Humanities, University of Glasgow, Glasgow, G12 8QQ UK

**Keywords:** Fundamentality, Grounding, Truthmaker semantics, Humeanism

## Abstract

Fundamentality plays a pivotal role in discussions of ontology, supervenience, and possibility, and other key topics in metaphysics. However, there are two different ways of characterising the fundamental: as that which is not grounded, and as that which is the ground of everything else. I show that whether these two characterisations pick out the same property turns on a principle—which I call “Dichotomy”—that is of independent interest in the theory of ground: that everything is either fully grounded or not even partially grounded. I then argue that Dichotomy fails: some facts have partial grounds that cannot be complemented to a full ground. Rejecting Dichotomy opens the door to recognising a bifurcation in our notion of fundamentality. I sketch some of the far-reaching metaphysical consequences this might have, with reference to big-picture views such as Humeanism. Since Dichotomy is entailed by the standard account of partial ground, according to which partial grounds are subpluralities of full grounds, a non-standard account is needed. In a technical “Appendix”, I show that truthmaker semantics furnishes such an account, and identify a semantic condition that corresponds to Dichotomy.

The question of what is fundamental in the world is of intrinsic interest. Moreover, an answer to it can guide and constrain what we say about other central metaphysical topics.

In discussions of *ontology*, it is a common view that the fundamental needs to be considered as carrying a heavy weight, while the non-fundamental can taken to be light-weight. Such a differential treatment may be articulated in a variety of ways: as the claim that fundamental properties are universals and non-fundamental ones mere classes (Armstrong 1978; Lewis 1983); or that fundamental facts are states of affairs and non-fundamental facts mere propositions (Armstrong 1997); or as the general methodological maxim that Ockham’s razor should be wielded only against putative fundamental entities (Schaffer 2015).

In discussions of *supervenience*, it is a common view that the non-fundamental supervenes on the fundamental: the former is fully modally fixed by the latter. Thus David Lewis:

I hold, as an *a priori* principle, that every contingent truth must be made true, somehow, by the pattern of coinstantiation of fundamental properties and relations. The whole truth about the world $\ldots$ supervenes on this pattern. (Lewis 1999, 292)

The roles of fundamentality vis-à-vis ontology and vis-à-vis supervenience are related. One obvious bridge between them is provided by Armstrong’s doctrine of the ontological free lunch: “what supervenes is no addition to being” (Armstrong 1997, 12).

In discussions of *possibility*, it is a common view that the fundamental entities are recombinable with each other:

[T]he fundamental actual concrete objects should be *freely recombinable*, serving as independent units of being $\ldots$. Thus each should be, in Hume’s words, ‘entirely loose and separate’. (Schaffer 2010, 40)

[W]hatever the (contingent) fundamental elements of the world are, they are open to free modal recombination. (Bennett 2017, 190)

The roles of fundamentality vis-à-vis supervenience and vis-à-vis possibility complement each other in fully determining the space of possibilities: the possibilities are all and only the recombinations of the fundamentalia.

The question of what is fundamental raises a prior question, which I shall be focussing on here: what is it for a fact, object, or property to be fundamental? It is natural to take fundamentality to be a matter of something’s place in the structure of the world. Such structure is, in turn, naturally articulated in terms of a certain relation. Indeed, once we have a term for such a relation—“ground”, by now entrenched in philosophical discourse—then using it to characterize fundamentality may not merely be optional, but required by the principle of ideological parsimony.^1^

What is fundamental may also be called the “foundations.” In a house, the foundations can either be described in terms of what is below them—namely, no other part of the house—or in terms of what is above them—namely, every other part of the house. Since we are accustomed to think of grounds as lower than what they ground, there are, correspondingly, two obvious strategies for defining the fundamental in terms of ground: as that which is ungrounded, or as that which grounds everything else. I shall say that these are, respectively, characterizations of the fundamental as the “ungrounded” and as the “all-grounding.”

Perhaps the structure of reality is not given just by one relation, but rather by a family of relations which are not usefully amalgamated into one: the “building relations”, in the terminology of Karen Bennett (2017). If so, the same strategies for defining fundamentality remain available, as long as each of these relations is suitably vertically directed, with the relata on one side being thought of as lower than those on the other side. The fundamental can then be thought of as the “unbuilt” or as the “all-building.” This is so regardless of whether ground itself is or is not among the building relations, as in the views of Bennett and of Jessica Wilson (2014), respectively.^2^

As I noted above, fundamentality is supposed to play a certain theoretical role in discussions of ontology, supervenience, and possibility. Between them, the two characterisations seem to account for its fitness to play that role. That the fundamental is ungrounded explains why it should be taken to be ontologically weighty, and why the fundamentalia do not modally constrain each other; that the fundamental is all-grounding explains why the non-fundamental need not carry an ontological cost, and why the fundamentalia modally constrain the non-fundamentalia.

Both styles of characterization are ubiquitous in the literature. The implicit assumption seems to be that they pick out the same notion. But the tenability of this assumption is underexplored. Bennett and Michael Raven (2016) deserve credit for contrasting the two characterisations explicitly, and exploring how they are related. In Sect. 1, I shall extend one of Bennett’s results. This discussion sets the stage for the main thesis of this paper: that the two characterisations may diverge.

My key move involves rejecting the orthodox account of the relationship between the notions of full ground and partial ground: that something is a partial ground iff there is something else together with which it is jointly a full ground. It follows from this that everything is either fully grounded or not even partially grounded—a principle I shall call *Dichotomy*. I give reasons to think that Dichotomy is false, and that the orthodox account of partial ground should be rejected—a thesis that is of independent interest for the theory of ground. If I am right, the notion of fundamentality bifurcates. Some potential metaphysical consequences of this bifurcation are sketched in Sect. 5. In the technical “Appendix”, I identify a mereological condition that corresponds to Dichotomy in Kit Fine’s truthmaker semantics for ground.

## Two conceptions of the fundamental

If something is ungrounded, I shall call it “B-fundamental”. (The letter B may be taken to stand for “basic”, but this no more than a memory aid.) This technical term is defined as follows:

- *f* is B-fundamental =$_{df}$ there is no full ground for *f*.

An entity is thus B-fundamental just in case it is at the bottom of the hierarchy generated by the relation of ground.

B-fundamentality may then be proposed as an explication of the notion of fundamentality. On the face of it, this is a plausible proposal, and does not call for extensive motivating commentary. I shall discuss its merits later, by comparing and contrasting it to a rival explication.

I noted in the introduction that we might wish to characterize the structure of the world using several relations—the building relations—rather than the one relation of ground. If something is not built by any relation, Bennett calls it “independent”. To avoid the ordinary connotations of that term, I shall call it B-fundamental* instead:^3^

- *f* is B-fundamental* =$_{df}$ there is no $\Gamma$ and building relation *R* such that $R(\Gamma ,f)$.

If ground is a building relation, then everything that is B-fundamental* is B-fundamental. If ground is the only building relation, then the converse is true as well.

I shall now turn to the second conception of the fundamental, as the all-grounding. The idea is, roughly, that the fundamental entities are such that everything in the world can be accounted for in terms of them—that they are “complete”, in Bennett’s terminology. In my further discussion, I shall take the fundamentalia to be facts. However, much of the discussion will carry over, *mutatis mutandis*, to entities in other ontological categories.^4^

It is tempting to say that some facts are all-grounding iff every fact is grounded in some of them. But there cannot be any such facts, provided some fact is B-fundamental. A remedy that suggests itself is to make an exception for the fundamental facts themselves—they need not be grounded, either by themselves or by any other facts. So we could say that the fundamental facts are those that provide a ground for every non-fundamental fact. However, this clearly cannot serve as a definition of fundamentality: as such, it would be circular.

To obtain a more careful formulation of the characterization of the all-grounding, I shall introduce a ground-theoretic analogue of the familiar notion of a supervenience base. Say that $\Gamma$ is a *grounding base* iff for every *f* that does not belong to $\Gamma$, there is $\Gamma ' \subseteq \Gamma$ such that $\Gamma '$ is a ground for *f*.^5^

Being a grounding base is a good explication for what it is for some facts to be complete, and thus to be candidates for being the fundamental facts. However, it is a condition that the fundamental facts need to satisfy collectively, and does not give us an explication of *f*’s being a fundamental fact without further work.^6^

It will not do say that *f* is fundamental iff it belongs to some grounding base. Since the plurality of all facts vacuously satisfies the definition of being a grounding base, that proposal would count everything as fundamental.

A more promising suggestion results from universally rather than existentially quantifying over grounding bases (with ‘A’ suggesting “all-grounding”):

- *f* is A-fundamental =$_{df}$*f* belongs to every grounding base.

The idea is that whatever full account of the world we offer, *f* will be an indispensable part of that. Using terminology from Raven (2016, 609), we might also say that *f* is “fundamental *qua* ineliminable”. I shall adopt A-fundamentality as my characterization of the fundamental in terms of all-grounding. (I shall say a bit more about this choice in the final two paragraphs of this section.)

I have now offered two definientia for *f*’s being a fundamental fact. This prompts the question how they are related. When discussing this, I shall appeal to the following standard principle:^7^

Irreflexivity $$\begin{aligned} A \text { does not belong to any ground for }A. \end{aligned}$$

Bennett (2017, 112) shows that B-fundamentality entails A-fundamentality—that “[e]very independent entity is in every complete set”. As it turns out, the converse entailment holds as well, such that our two notions are equivalent:

### **Proposition 1**

*Suppose that ground is irreflexive. Thenfis B-fundamental*$$\Gamma$$$$\Leftrightarrow$$*fis A-fundamental*.

### *Proof*

$\Rightarrow$: Suppose that *f* is not A-fundamental. Then there is some grounding base $\Gamma$ to which *f* does not belong. It follows that there is $\Gamma ' \subseteq \Gamma$ such that $\Gamma '$ is a ground for *f*. Hence *f* is not B-fundamental.

$\Leftarrow$. Suppose that *f* is not B-fundamental. Hence there is a ground $\Gamma$ for *f*. By Irreflexivity, $f \not \in \Gamma$. Now let $\Delta$ be all the facts. Clearly, $\Delta$ is a grounding base. Consider $\Delta \setminus \{f\}$ (i.e. the set-theoretic difference of $\Delta$ and $\{f\}$), and let *g* be an arbitrary fact. If $f = g$, then $\Gamma \subseteq \Delta \setminus \{f\}$ is a ground for *g*. If $g \ne f$, then $g \in \Delta \setminus \{f\}$. So $\Delta \setminus \{f\}$ is a grounding base. That is, *f* does not belong to every grounding base, and is not A-fundamental. $\square$

The result that the two characterizations coincide is robust: it continues to hold if we let fundamentality depend on several building relations, rather than the one relation of ground, as I shall now show.

To generalize A-fundamentality, we first introduce the notion of a building base. Say that $\Gamma$ is a *building base* iff for every *f* that does not belong to $\Gamma$, there is $\Gamma ' \subseteq \Gamma$ and a building relation *R* such that $\Gamma '$ stands in *R* to *f*.^8^

We can then define:

- *f* is A-fundamental* =$_{df}$*f* belongs to every building base.

Notice that the definitions of the starred concepts are like those of the unstarred ones, except that the role of ground is now played by the disjunction of all building relations—the relation $\bigvee \mathcal {B}$ that holds between $\Gamma$ and *f* just in case there is some *R* among the building relations $\mathcal {B}$ such that $\Gamma$ stands in *R* to *f*. That relation $\bigvee \mathcal {B}$ may well fail to satisfy the criteria for being a building relation. For example, it may not be asymmetric even if all building relations are asymmetric, and it may not be transitive even if all building relations are transitive. But provided that every building relation is irreflexive, $\bigvee \mathcal {B}$ is irreflexive as well. The proof of Proposition 1 thus straightforwardly carries over to yield a generalized result:

### **Proposition 2**

Suppose that every building relation is irreflexive. Thenfis B-fundamental* $$\Gamma$$$$\Leftrightarrow$$fis A-fundamental*.

Propositions 1 and 2 only rely on the assumption of irreflexivity. Other standard assumptions, such as asymmetry or transitivity, are not needed; nor is the assumption that chains of ground end in ungrounded facts. Even if there is an “infinite descent” of ground, then B-fundamentality and A-fundamentality coincide.^9^ However, it needs to be acknowledged that A-fundamentality may not be a good explication of fundamentality in terms of the all-grounding in such a situation: the A-fundamental things will not, collectively, be a grounding base. Each of them will be ungrounded, but they will not be jointly all-grounding. 

It is not clear what should count as fundamental in the sense of all-grounding in a world of infinite descent.^10^ Perhaps the most plausible thing to say is that the notion is not defined relative to such a world. At any rate, I shall set such scenarios aside for the rest of this paper. Many philosophers take them to be incoherent anyway, and will not object to this restriction.^11^ Fans of infinite descent may already be open to the idea that characterizations of the fundamental in terms of being ungrounded and being all-grounding may diverge. But even for them, there is something new in the rest of the paper: the claim that the two characterizations can come apart even in the absence of infinite descent; and that they can come apart even though both are defined, rather than one being defined and the other not.

## Disambiguating definitions of fundamentality

A-fundamentality and B-fundamentality are stated using a generic notion of ground. Among the various distinctions in the family, at least one deserves attention in the present context: that between full ground and partial ground. I will discuss this distinction in more detail in a subsequent section; for now I only assume that ‘partial ground’ is used inclusively, to apply to full grounds too.

When we characterise the fundamental as the ungrounded, we presumably have the notion of partial ground in mind: only facts that are not even partially grounded are wholly ungrounded. It is natural to relate fundamentality as ungroundedness to a certain hierarchical conception of the world: an entity is fundamental, in the relevant sense, if and only if nothing is below it in the hierarchy. But this hierarchical structure of the world is typically articulated by the relation of partial ground—a binary relation that we can represent in a graph. (The relation of full ground, being plural on the left-hand side, does not lend itself to convenient pictorial representation.)

When we characterise the fundamental as the all-grounding, in contrast, we presumably have the notion of full ground in mind. In metaphysics, we are interested in identifying collections of facts which are complete, in the sense of accounting for everything. As the term ‘complete’ suggests, we are looking for *full* grounding bases—where ‘ground’ in the definition of a grounding base is read as ‘full ground’. The fundamentalia should provide more than merely partial grounds for everything else. This seems to be required by the theoretical role of fundamentality vis-à-vis supervenience: why should a mere partial grounding base modally fix everything else?

To relate this discussion with our previous results, it will be useful to extend our terminology. I shall say that *f* is *strongly* B-fundamental iff no $\Gamma$ partially grounds it; and *weakly* B-fundamental iff no $\Gamma$ fully grounds it.^12^ Since any full ground of a fact is also partial ground, strong B-fundamentality entails weak B-fundamentality, as we might expect. 

Further, I shall say that *f* is *weakly* A-fundamental iff it belongs to every full grounding base; and *strongly* A-fundamental iff it belongs to every partial grounding base. Since full grounds are partial grounds, every full grounding base is a partial grounding base, and hence strong A-fundamentality entails weak A-fundamentality. While the natural notion of completeness, which provides a constraint on the fundamentalia collectively, is the stronger one—being a full grounding base—membership in every strongly complete base is a weaker condition than membership in every weakly complete one.

**Fig. 1:**
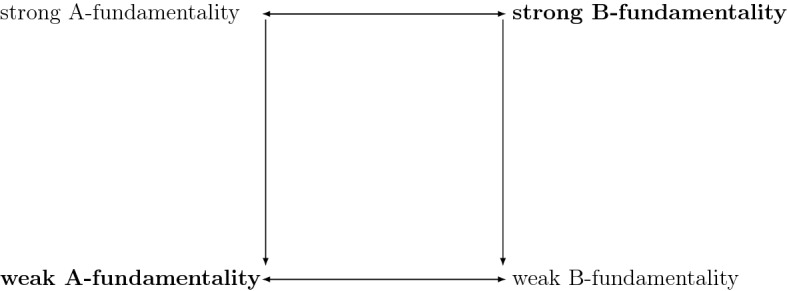
Four candidate explications of fundamentality

The mutual entailment of strong B-fundamentality and strong A-fundamentality, as well as of the corresponding weak notions, are consequences of Proposition 1. The logical relationship between our four notions are summarised in Fig. 1, with arrows symbolizing logical entailment (on the assumption that full ground and partial ground are both irreflexive).

The natural notions of fundamentality for individual facts are strong B-fundamentality and weak A-fundamentality—marked in bold in the figure. Inspection of Fig. 1 confirms that strong B-fundamentality entails weak A-fundamentality. However, the converse entailment has not been shown to hold. In the rest of the paper, I shall discuss under what conditions it does hold.

## Full ground and partial ground

One of our candidate explications of fundamentality—strong B-fundamentality—is defined in terms of full ground, and the other—weak A-fundamentality—in terms of partial ground. We need to take a closer look at these relations.

The distinction between full grounds and partial grounds does not map neatly to the various words in philosophers’ English that the idiom of ground is supposed to regiment—‘in virtue of’, ‘because’, ‘explains’, ‘accounts for’ and their cognates may all be used to express claims of partial or of full ground.^13^ The contrast is analogous to the one between a partial cause—which is often simply called “cause”—and a full cause—something that is typically too complex to cite.^14^ The distinction can be elucidated by examples. Let us suppose that Anna’s being my niece is grounded in Martin’s being my brother and Anna’s being Martin’s daughter. This is a case of full ground: the grounds fully account for what is grounded. It also makes sense to say that my being an uncle is grounded in Anna’s being Martin’s daughter. But here, we would have a case of partial ground: the ground contributes to what is grounded. Furthermore, it is a case of *merely partial* ground: the partial ground is not also a full ground. Likewise, my having a mass is a partial ground for my attracting the earth. But it is a merely partial ground, since a full ground would also include further facts, for example the earth’s having mass and the holding of the law of gravitational attraction.

There is a reason why in ordinary usage, locutions in the relevant family are often indicative of a connection of partial ground only. Once we go beyond toy examples like the above, we would often find it hard to specify full grounds. What is the fact that France is a nation grounded in, for example? Presumably, we could all cite, or at least gesture at, several partial grounds—facts about its internal organization, about international law, or, at a deeper level, about the intentions of its citizens. But it would be very difficult indeed to cite any full grounds—especially so if necessitarianism about full ground holds, that is, if the conjunction of the grounds needs to strictly imply what is grounded. For then the grounds will typically also include an indefinite number of facts relating to background conditions, or about the absence of potentially interfering factors. But even on a non-necessitarian conception, full grounds are often too large to specify.

As has often been remarked, the notion of ground is closely related to that of metaphysical explanation. We are certainly familiar with the concept of a partial explanation. When we ask for an explanation of some fact, we may get an answer that partly satisfies what we are asking for. (Indeed, we typically do not request more than a partial explanation in the first place.) A merely partial ground does not fully account for the fact, but goes some way towards it. Or, to use another paraphrase, we can say that to be a partial ground for a certain fact is to contribute to its holding.

On what is arguably the orthodox account, put forward by Correia (2005, p. 60) and Rosen (2010, p. 115) among others, partial ground is defined in terms of full ground. In a slogan: a partial ground is a part of a full ground. Usually, ‘part’ is not understood in the strict mereological sense here. Rather, something is a partial ground if and only if it is one of some things that are, together, a full ground. Or to put it differently: to a be a partial ground is to be completable to a full ground.

Presumably, such a definition is not intended to be merely stipulative, concerning the use of a technical term. As I pointed out, many pertinent ordinary locutions are supposed to express partial ground. I shall thus treat the orthodox account as asserting a substantive claim about the relationship between full ground and partial ground.

The account is usefully broken down in two claims: that partial grounds are *extractable*—being part of a full ground is sufficient for being a partial ground—and that partial grounds are *completable*—being part of a full ground is necessary for being a partial ground. I shall not discuss the extractability condition.^15^ My concern will be with the question whether partial grounds are completable.

In fact, I shall mainly focus my discussion on the following consequence of the claim that every partial ground is completable:

Dichotomy $$\begin{aligned} \text {If }f\text { has a partial ground, it has a full ground.} \end{aligned}$$

Or in the terminology introduced earlier: if *f* is weakly B-fundamental, then it is strongly B-fundamental.

If ground is dichotomous, then all of our four notions of fundamentality coincide, as inspection of the figure reveals. In particular, it will turn out that the explications of the fundamental as the ungrounded and as the all-grounding boil down to the same thing.

## Merely partially grounded facts?

In general, a partial *F* need not be a part of a full *F*. If my employer gives me a partial reimbursement for my expenses, it does not follow that someone else will reimburse the remainder, alas. Likewise, the world is full of examples of partial success in the absence of full success. The question then is whether in the specific case where *F* is ‘ground’—more precisely, ‘ground for *g*’, for some given *g*—there always needs to be a full *F*.

Completability has been largely unchallenged in the literature on ground. The following suggestive passage by Kit Fine is a notable exception:

[There is] a natural partial notion of ground for which a partial ground need not always be part of a full ground. One might wish to say, for example, that the truth that P is a partial ground for knowledge that P, even though there is nothing one might add to P to obtain a strict full ground for knowledge that P (as in the view of Williamson 2000). (Fine 2012a, p. 53)

I shall not discuss whether the truth of P should indeed count as a non-completable partial ground of knowledge that P, on Williamson’s theory. Whether it is or not, it seems to me that the “knowledge first” theory does not provide us with a counterexample to Dichotomy.^16^ Certain facts about Fred’s brain, about his environment, and about how he causally relates to the environment are modally sufficient for Fred’s knowing that P.^17^ While Williamson does not use the idiom of ground himself, it is natural to say that those facts, or perhaps some relevant subclass thereof, jointly ground Fred’s knowing that P. What Williamson denies is that any (possibly disjunctive) brain conditions, or any (possibly disjunctive) environmental conditions, are necessary as well as sufficient for knowing that P. His claim is that knowing that P cannot be *analysed* with P as a conjunct, not that facts involving that property are ungrounded.^18^

Similar remarks apply to cases that involve causation or action rather than knowledge. That *c* and *e* both happen may be a non-completable partial ground of *c*’s causing *e*; and that my arm goes up may be a non-completable partial ground of my raising my arm. In neither case, however, do we have good reason to think that there are no full grounds of the relevant facts.

I am, however, sympathetic to the idea that Dichotomy fails, and shall now sketch two potential counterexamples. The first one involves an imaginary scenario, and does not rely on specific metaphysical commitments; the second one turns on certain theoretical ideas about totality.

Consider the following scenario. In worlds $w^+$ and $w^-$, a determinable quality, to be called “schmarge”, is instantiated. Schmarge is akin to electric charge in displaying a positive–negative polarity. The space of its determinates is simpler, though—it has only two rather than infinitely many determinates. We may call them “positive schmarge” and “negative schmarge”, but like in the case of electric charge, these labels are entirely arbitrary. Positive schmarge is no more similar to positive charge, or to positive growth, than it is to negative charge, or negative growth, respectively.

In worlds $w^+$ and $w^-$, there are no infinitely descending chains of ground. Moreover, those worlds are mereologically atomic. Atoms tend to cluster in stable aggregates, which we may call “molecules”. The laws of nature of $w^+$ and $w^{-}$ are such that a molecule has schmarge if and only if it is composed of an even number of atoms, all of which have the property *F*. That property is fundamental in both worlds, and every atom that has it does so contingently. Among the molecules with schmarge, some are positive, and some negative. However, for any given molecule with schmarge, it is a brute fact what its polarity is. There is no law linking its polarity with its composition, or with any of its other intrinsic features. (To be sure, there is a law linking the distribution of positive and negative schmarges with a field of intermolecular forces, but that field is generated by the positive and negative schmarges, rather than explaining them.)

Positive and negative schmarge exhibit a distinctive failure of supervenience. In $w^+$, molecule *a* has positive schmarge. But in world $w^{-}$, *a* has negative schmarge. Yet every fundamental fact of $w^+$ obtains, and is fundamental, in $w^-$—except possibly the fact that *a* has positive schmarge, whose status as fundamental or non-fundamental shall not be pre-judged. Likewise, every fundamental fact of $w^{-}$ obtains, and is fundamental in $w^+$—except possibly the fact that *a* has negative schmarge.

*Prima facie*, the schmarge scenario is metaphysically possible. Positive and negative schmarge do not supervene on the fundamental properties of their atomic parts, but their instantiation by a whole still has a certain configuration of the parts as a necessary condition.

It seems to me that the scenario describes a plausible possible counterexample to the dichotomy of ground. When describing it, I took care not to pre-judge the question whether $[G^+a]$, the fact that *a* instantiates positive schmarge, is grounded in something, and if so, by what. We shall now ask that question.

It seems to me that $[G^+a]$ does not have any full grounds. There do not appear to be any suitable candidates around to play that role. Yet I would argue that $[G^+a]$ is partially grounded. Let [*Ha*] be the fact that *a* is composed of an even number of particles each of which has *F*. It is then plausible that in world *w*, $[G^{+}a]$ is partially grounded in [*Ha*], or perhaps by [*Ha*] together with a pertinent fact *l* concerning the laws of nature of *w*. After all, [*Ha*] and *l* together go some way towards explaining $[G^{+}a]$; and it is natural to say that $[G^{+}a]$ obtains because [*Ha*] does, or in virtue of [*Ha*] obtaining.^19^

So we have at least one example where Dichotomy plausibly fails. To warm up for the second potential example, which involves ideas about totality, ponder proposition 1.11 of the *Tractatus*:

The world is determined by the facts, and by these being all the facts.

The addition of “these being all the facts” is prompted by the need to account for some negative and universally quantified truths. On one way of spelling out this idea, there is a plurality $\Gamma$ of *first-order* facts, and there is also a further fact *t* (for “totality”): that $\Gamma$ are all the first-order facts. The totality fact *t* thus entails each one of the facts in $\Gamma$.

According to the most developed account of this kind, presented in D. M. Armstrong (2004, 72), the fact *t* involves a relation of *alling* or *totalling*. Specifically, it is the fact that the mereological fusion of all first-order facts stands in that relation to the property of being a first-order fact.^20^

Supposing that this is correct, we can now ask what, if any, relationships of ground obtain between $\Gamma$ and *t*.

The view under consideration belongs to the logical atomist tradition. The facts in $\Gamma$ are taken to be logical atoms. In the key of ground, this can be expressed by saying that they are ungrounded. Specifically, none of them is grounded in *t*, either fully or partially.

Even if $\Gamma$ consists of all the first-order facts, it is possible for all the facts in $\Gamma$ to obtain without *t*: there could be further first-order facts, after all. This point was emphasized by Russell, and it presumably prompted Wittgenstein to add “and by these being all the facts” to “the world is determined by the facts”. On the widely held assumption that grounds necessitate what they ground, it cannot be the case that $\Gamma$ is a full ground for *t*.

What is left open so far is whether $\Gamma$ is a partial ground for *t*. It seems to me that it is: *t* is a complex fact which entails the obtaining of all members of $\Gamma$; indeed, the members of $\Gamma$ are naturally taken to be constituents of the complex fact.^21^ Hence *t* holds in part because the facts in $\Gamma$ do, or in virtue of the facts in $\Gamma$ obtaining.

But in the ontology in question, there is no plausible candidate for a full ground of *t*. We have seen that $\Gamma$ is not—this is the reason why the totality fact has been introduced in the first place. Moreover, apart from *t* and the members of $\Gamma$, there is not really anything else in the logical atomist’s ontology.^22^

This second potential counterexample to Dichotomy could be resisted by adopting a different, purely exclusive account of the totality fact: as the fact that *at most* the actual first-order facts obtain—a fact that does not entail any of them, and is arguably not partially grounded in any of them. We need to note, though, that this is indeed a different conception of totality facts than the inclusive one that is adopted in the relevant literature. Moreover, the change in conception may incur costs. When discussing the two conceptions, Kit Fine notes that he is “inclined to prefer [the inclusive conception] on theoretical grounds” (2012a, 62).^23^ Fine’s and Armstrong’s theoretical considerations—discussion of which would lead us too far afield—may or may not turn out to be weighty. The important point, though, is that if their account of totality were rejected to save Dichotomy, that principle would turn out to have revisionary consequences. This confirms my suspicion that the standard account of partial ground, which entails Dichotomy, is not a harmless regimentation of our talk of ground. Rather, it embodies substantive metaphysical commitments—a theme I shall take up again in the next section.

I started this section by reporting a putative counterexample to Dichotomy due to Kit Fine. While I do not find the example itself convincing, I wish to draw on Fine’s work to bolster my claim that Dichotomy may fail. Fine suggests his example in the course of distinguishing various different notions of partial ground that are definable in his semantics. At a minimum, his framework can be used to show that a notion of partial ground that violates Dichotomy is logically well-behaved.

The semantics involves some entities—“verifiers” or “truthmakers”—standing in mereological relations. As I show in the “Appendix”, Dichotomy is closely related to the holding of the so-called “Weak Supplementation” principle among the verifiers. Such a principle rules out models, *inter alia*, where something has just one proper part. The totality fact *t*, in the above example, is naturally taken to have a verifier which has the fusion of $\Gamma$, the first-order facts, as a proper part, but no parts which do not overlap that fusion.

If we are prepared to take the semantics with metaphysical seriousness, rather than being instrumentalist about it, we can mine the literature on mereology to construct further potential counterexamples to Dichotomy. While Weak Supplementation is widely accepted, there is also notable precedent for rejecting it. Franz Brentano (1981, 47) thought that the thinking soul has the soul as a part, but is not the result of the soul “acquiring a second part.” He holds the same about what he calls “logical parts”:

When we compare “red thing” and “colored thing” we find that the latter is contained in the former, but we cannot specify a second thing that could be added to the first as an entirely new element, i.e., one that would not contain the concept, colored thing. (1981, 112)

Another precedent is provided by Whitehead, on whose theory open interiors are proper parts of topologically closed regions, even though there are no boundaries which could serve as supplements.^24^

In the more recent literature, further potential counterexamples to Weak Supplementation have been explored. It would be beyond the scope of this article to survey that work.^25^ Some of these examples might be adapted to challenge Dichotomy directly; others indirectly, via Fine’s mereological models for ground discussed in the “Appendix”.

## Weak and strong fundamentality

If I am right that Dichotomy may fail, we need to distinguish between weak and strong fundamentality. While it is beyond the scope of the present paper to explore the ramifications of such a move, I shall close by sketching one way in which it might be significant.

Weak and strong fundamentality will split the theoretical role described at the beginning of this article. Weak fundamentality constrains ontology: accepting only the strongly fundamental entities is not enough to meet one’s commitments. Likewise, it is weak fundamentality that limits modal recombination: everything supervenes on the weakly fundamental. In contrast, it is strong fundamentality, at best, that guarantees modal recombination.

Recognising that these roles are played by different properties may prompt us to reassess a number of metaphysical theses and arguments. Consider Humeanism in metaphysics. To a first approximation, contemporary Humeanism holds that there is a modally recombinable basis for everything. The paradigmatic representative is David Lewis, whose “pointillism” takes the recombinable basis to consist of instantiations of perfectly natural qualities at spacetime points. But the view comes in a number of different versions. The members of the basis may be objects, or properties, or facts, or entities of yet another category. The key relation between the basis and the rest may be supervenience, truthmaking, dependence, ground, building, or yet something else. Some view in the vicinity of Humeanism is shaping the view of a number of contemporary metaphysicians, regardless of whether they label their view as “Humean”.

The principle that the members of the basis are recombinable is a powerful one: it has been used to argue that there is no such thing as a structural universal (Lewis 1986), or a totality state of affairs (Cameron 2010), and that facts involving the building relations are themselves built (Bennett 2017, 190). Moreover, the principle has played a key role in recent arguments for prioriy monism, the view that one and only entity is fundamental (Cameron 2010; Schaffer 2010). Priority monism is compatible with the principle: if modal recombinability is just the lack of necessary connections among distinct members of the basis, then a basis consisting of one element is trivially recombinable. Its proponents argue that given certain other plausible background views, it is the only view compatible with it.^26^

I shall not discuss whether Humeanism does indeed have the implications it is claimed to have. Rather, my interest is in the status of Humeanism itself—a view more often assumed than argued for. What might motivate it? It is not hard to see that fundamentality might figure as a middle term in an argument for it.^27^ For specificity, take Humeanism to be the view that there is a recombinable grounding base. Then the view is a consequence of the following two premises:

1. The fundamental facts are modally recombinable.
2. The fundamental facts form a grounding base.

We can see this line of reasoning as a master argument for Humeanism. I suspect that it plays an important, though mostly implicit, role in motivating the view. Perhaps the most explicit argument in this form is given in Schaffer (2010). Schaffer is concerned with objects rather than facts, and talks of “basic” rather than “fundamental” entities. He defines a concrete object as *basic* iff it does not depend on anything (38). He also points out, plausibly enough, that “[i]f entities are metaphysically independent, then they should be modally unconstrained in recombination” (40). So his basic entities are modally recombinable—a variant of (1) above. His crucial move is to requires that “the basic entities must be complete, in the sense of providing *a blueprint* of reality” (39)—a variant of (2). He concludes that the basic objects form a modally recombinable dependence basis, a thesis which entails a version of Humeanism.

But if the argument of this paper is right, premise (1) in the master argument for Humeanism plausibly holds if we are talking about B-fundamentality, and premise (2) if we are talking about A-fundamentality. For the argument to go through, B-fundamentality and A-fundamentality need to coincide. But they may not, as I have argued in this paper. If that argument has succeeded, Humeanism is no longer mandatory, and the set of options for our fundamental world view has correspondingly expanded.

## Appendix: Failures of Dichotomy in mereological frames

In his 2012a, b, Kit Fine gives a semantics for ground in terms of sets of “states”, “verifiers” or “truthmakers” that stand in mereological relations. This “Appendix” investigates what mereological conditions correspond to the failure of Dichotomy.

Fine presents his mereology in a guise that may look unfamiliar to many metaphysicians. First, he assumes the existence of a “null individual” 0, which is part of everything. Such a putative entity has typically been shunned by mereologists. I will follow Fine here, since his is the standard framework for this kind of semantics. But I wish to emphasize that my results do not depend on there being a null individual. Indeed, admitting 0 leads to a number of complications in my discussion.

Second, Fine’s notion of a fusion relates differently to parthood than either of the two notions of fusion that have currency in the metaphysical literature. For Fine, a fusion of some things is their least upper bound. If *t* has every member of *G* as a part and every part of *t* overlaps some member of *G*—one common notion of fusion—then *t* is a fusion of *G* in Fine’s sense; and if the mereology is distributive (Fine 2017, 646), the converse holds as well. Stronger assumptions would be needed to guarantee coincidence with *t*’s being a fusion of *G* in the sense that something overlaps *t* just in case it overlaps some member of *G*.^28^

Third, Fine (2012b) chooses a fusion function as his primitive, rather than parthood.^29^ Hence he does not require an axiom guaranteeing the existence of fusions. If $\bigsqcup G$ is the fusion of members of the set *G*, $\bigsqcup \{x,y\} = \bigsqcup \{y\}$ can be used to define the parthood relation $x \,\sqsubseteq \,y$ . However, along with Fine himself in a more recent piece of work (2017), I shall take parthood as primitive.

While Fine uses mereological frames to give a semantics for a family of binary sentential connectives, I shall use them to give a model—in the non-model-theoretic sense of the word—of facts and relations of ground that hold between them. The mereological frames can be used for either purpose, just like possible worlds frames can be used either to give a model theory for formal languages, or to give a model of propositions and certain relations like entailment and incompatibility between them.

A binary relation $\sqsubseteq$ is a *partial order* on a set *F* iff it is reflexive, transitive, and anti-symmetric. An element *y* is an *upper bound* of a subset *G* of *F* iff $x \sqsubseteq y$ for all $x \in G$; and it is a *fusion* or least upper bound of *G* iff it is an upper bound of *G*, and for every upper bound *z* of *G*, $y \sqsubseteq z$.

A *mereological frame*$\mathcal {F}$ is a pair $\langle F, \sqsubseteq \rangle$, where *F* is a set and $\sqsubseteq$ a partial order on *F* such that every $G \subseteq F$ has a fusion. Since $\sqsubseteq$ is anti-symmetric, every $G \subseteq F$ has a unique fusion $\bigsqcup G$. I shall also use the following symbols and definitions for the null element, proper parthood, the set of non-null proper parts, and overlap, respectively:

- $$0 := \bigsqcup \emptyset$$
- $$x \sqsubset y := x \sqsubseteq y \wedge x \ne y$$
- $$p(x) := \{z: z \sqsubset x \wedge z \ne 0\}$$
- $$Oxy := \exists x (z \ne 0 \wedge z \sqsubseteq x \wedge z \sqsubseteq y)$$

Fine (2012b) calls the members of *F* ‘facts’, and non-empty subsets of *F* that are closed, in the sense of including fusions of all of their non-empty subsets, ‘propositions’. These latter entities are eligible to be semantic values in his framework, and they are quantified over in the definition of ground. I shall diverge from his use, and call such subsets of *F* ‘facts’. Since we can disregard false propositions or non-actual verifiers in an application to the pure logic of ground—as opposed to certain other semantic phenomena Fine (2017) is interested in—there is nothing incongruous in my usage. While the question whether we should take facts to be the relata of ground is an important one, I take it to be largely orthogonal to the concerns of the present paper.

More precisely, I shall take facts to be those sets of truthmakers that are, in the terminology of Fine (2017, 647), neither vacuous nor trivial, and closed under fusion.

### **Definition 1**

A set $G \subseteq F$ is a *fact* iff *G* is non-empty, $0 \not \in G$, and for every non-empty $H \subseteq G$, $\bigsqcup H \in G$.^30^

For a set of facts $\Gamma$, let a *choice function* for $\Gamma$ be a function that maps every $g \in \Gamma$ to one of its members. (Notation: when a subset of *F* is also a fact, I sometimes denote it with a lower-case variable $f, g, \ldots$.) Since facts are non-empty, every set of facts has a choice function. Let a *choice* for $\Gamma$ be the range of a choice function for $\Gamma$.

Let $\Gamma$ be a set of facts, and *f* a fact.^31^

- $\Gamma$ is a *full proto-ground* of *f* iff for any choice *G* for $\Gamma$, $\bigsqcup (G) \in f$;
- $\Gamma$ is a *partial proto-ground* of *f* iff for some $\Delta$, $\Gamma \cup \Delta$ is a full proto-ground for *f*.^32^

We can now define partial ground and full ground:

- $\Gamma$ is a *partial ground* for *f* iff $\Gamma$ is a partial proto-ground for *f*, and *f* is not a partial proto-ground for any $g \in \Gamma$.
- $\Gamma$ is a *full ground* for *f* iff $\Gamma$ is a full proto-ground for *f*, and *f* is not a partial proto-ground for any $g \in \Gamma$.

I shall now investigate under what conditions the following principle, introduced in Sect. 3, holds in a mereological frame.

Dichotomy $$\begin{aligned} \text {If }f\text { has a partial ground, it has a full ground.} \end{aligned}$$

Say that *x* is *atomic* iff $x \ne 0$ and $p(x) = \emptyset$; and *x* is *properly fused* iff $x = \bigsqcup p(x)$. We note that an atomic *x* is not the fusion of $\emptyset$; hence it is not properly fused.

The following principle says that if something has a non-null proper part, it is properly fused:

Proper Fusion $$\begin{aligned} \forall x (\exists y (y \ne 0 \wedge y \sqsubset x) \rightarrow \bigsqcup p(x) = x) \end{aligned}$$

### **Proposition 3**

*If Proper Fusion holds in a mereological frame, so does Dichotomy*.

### *Proof*

Suppose that $\Gamma$ is a partial ground for *f*. Since *f* is a fact, it is non-empty and closed under non-empty fusions, such that $\bigsqcup f \in f$. We now show by *reductio* that $\bigsqcup f := x_f$ is not atomic. Suppose that it is. Then either $f = \{x_f\}$ or $f = \{x_f, 0\}$. Since *f* is non-trivial, $0 \not \in f$, and hence $f = \{x_f\}$. Given that for some $\Delta$, $\Gamma \cup \Delta$ is a full proto-ground for *f*, $\bigsqcup G \sqsubseteq x_f$ for every choice *G* for $\Gamma$. Since every member of $\Gamma$ is a fact and thus non-trivial, $0 \not \in G$. Hence $G = \{x_f\}$ and $\Gamma = \{f\}$. It follows that *f* is a full and thus a partial proto-ground for a member of $\Gamma$, contradicting the assumption that $\Gamma$ is a partial ground for *f*.

We now construct a full ground for *f*. Set $\Delta _f := \{\{x\}: x \in p(\bigsqcup f)\}$. That is, $\Delta _f$ consists of unit sets of non-null proper parts of the fusion of the members of *f*. We verify that all members of $\Delta _f$ are facts: as unit sets, they are non-empty, of course, and closed under non-empty fusions; and they do not contain 0 given the definition of the function *p*. Since $\bigsqcup f$ is not atomic, $\Delta _f$ is non-empty.

We first show that $\Gamma \cup \Delta _f$ is a full proto-ground for *f*. Consider any choice *G* for $\Gamma \cup \Delta _f$. Its fusion $\bigsqcup G$ is the fusion of a choice for $\Gamma$ and a choice for $\Delta _f$. Since $\Gamma$ is a partial ground for *f*, every member of the choice set for $\Gamma$ is a part of $\bigsqcup f$. As a set of unit sets, $\Delta _f$ has only one choice set $p(\bigsqcup f)$, each member of which is a part of $\bigsqcup f$. Hence $\bigsqcup f$ is an upper bound of *G*, such that $\bigsqcup G \sqsubseteq \bigsqcup f$. Moreover, we have $\bigsqcup p(\bigsqcup f) \sqsubseteq \bigsqcup G$. Since Proper Fusion holds, $\bigsqcup p(\bigsqcup f) = \bigsqcup f$, and hence $\bigsqcup f \sqsubseteq \bigsqcup G$. By anti-symmetry, $\bigsqcup f = \bigsqcup G$, and hence $\bigsqcup G \in f$.

It remains to show that there is no member of $\Gamma$ or $\Delta _f$ for which *f* is a partial proto-ground. Since by assumption $\Gamma$ is a partial ground for *f*, *f* is not a partial proto-ground for any $g \in \Gamma$. Consider any member $\{x\}$ of $\Delta _f$ and any $\Lambda$. Since $\bigsqcup f \in f$, $\{f\} \cup \Lambda$ will have a choice *G* with $\bigsqcup f \sqsubseteq \bigsqcup G$. Given that $x \sqsubset \bigsqcup f$, $x \ne \bigsqcup G$, and hence $\bigsqcup G \not \in \{x\}$. It follows that $\{f\} \cup \Lambda$ is not a full proto-ground for $\{x\}$, and thus that $\{f\}$ is not a partial ground for $\{x\}$. $\square$

(For those familiar with Fine’s terminology relating to the notion of partial ground: Proposition 3 entails that if Proper Fusion holds, then if *g* is a strict partial ground of *f*, it is also a partial strict ground of *f*. Since the converse holds without mereological assumptions, as Fine notes, it follows that the notions of strict partial ground and of partial strict ground coincide, given the assumption of Proper Fusion.)

We also get a necessary condition for Dichotomy to hold:

### **Proposition 4**

*If**x**is non-atomic but not properly fused, then**p*(*x*) *is a partial ground for*$\{x\}$, *but nothing is a full ground for*$\{x\}$.

### *Proof*

We first verify that *p*(*x*) is a fact. By definition, $0 \not \in p(x)$. Since *x* is non-atomic, either $x = 0$ or $p(x) \ne \emptyset$. But 0 is properly fused: $0 = \bigsqcup p(0)$. Since *x* is not properly fused, $x \ne 0$, and hence *p*(*x*) is not empty. To show that *p*(*x*) is closed under non-empty subsets, let $H \subseteq p(x)$. Since *H* is non-empty, $\bigsqcup H \ne 0$. Since *x* is an upper bound of *p*(*x*), it is an upper bound of *H*; and since $\bigsqcup H$ is the least upper bound of *H*, $\bigsqcup H \sqsubseteq x$. But since *x* is not properly fused, $x \ne \bigsqcup p(x)$ and hence $x \ne \bigsqcup H$. Hence $\bigsqcup H \sqsubset x$.

It is easy to see that $\{x\}$ is also a fact. Since the fusion of any choice for $p(x) \cup \{x\}$ is *x*, $p(x) \cup \{x\}$ is a full proto-ground for $\{x\}$. Since no *y* with $x \sqsubseteq y$ belongs to *p*(*x*), $\{x\}$ is not a partial proto-ground for *p*(*x*). This establishes that *p*(*x*) is a partial ground for $\{x\}$.

However, nothing is a full ground for $\{x\}$. Suppose that $\Gamma$ is a full proto-ground for $\{x\}$, and let *G* be any choice for $\Gamma$. Then $\bigsqcup G = x$. Hence any member of any $h \in \Gamma$ is a part of *x*. Since *x* is not properly fused, *G* includes a part of *x* that is not a proper part of *x*. Hence $x \in G$, and thus $x \in h$ for some $h \in \Gamma$. It follows that $\{x\}$ is a full, and thus a partial, proto-ground for *h*. Hence $\Gamma$ is not a full ground for $\{x\}$. $\square$

Hence we obtain a mereological condition that is necessary and sufficient:

### **Corollary 1**

*Dichotomy holds in a mereological frame iff Proper Fusion does*.

### *Proof*

If Proper Fusion holds, then so does Dichotomy, by Proposition 3. For the other direction, suppose that Proper Fusion is false. Then there is a non-atomic *x* that is not properly fused. By Proposition 4, *p*(*x*) is a partial ground for $\{x\}$, but nothing is a full ground for $\{x\}$. Hence Dichotomy fails. $\square$

I noted that admitting 0 leads to complications in my discussion. Definition 1 has the consequence that $\{0\}$ is not a fact. This is crucial for our result to hold. For suppose that $\{0\}$ is a fact. Then it is a partial proto-ground of every fact, and a partial ground of every fact distinct from itself. If *x* is atomic, then $\{x\}$ has no full ground, but it has $\{0\}$ as a partial ground, falsifying Dichotomy. Our results still hold, however, if we allow facts to include 0, as long as they do not only include 0. That is, they hold if a set is defined to be fact iff it is non-empty, closed under non-empty fusions, and distinct from $\{0\}$.^33^

The notion of something’s being properly fused is not one among those discussed in the mereological literature, to the best of my knowledge.^34^ I shall now relate Proper Fusion to another condition that is more familiar to metaphysicians: Weak Supplementation. In the presence of a null element, this can be stated as follows (Hovda 2009, 74):^35^

Weak Supplementation $$\begin{aligned} \forall x \forall y (y \ne 0 \wedge y \sqsubset x \rightarrow \exists z (z \ne 0 \wedge z \sqsubseteq x \wedge \lnot Oyz)) \end{aligned}$$

### **Proposition 5**

*If Weak Supplementation holds in a mereological frame, so does Proper Fusion*.

### *Proof*

Suppose $y \ne 0$ and $y \sqsubset x$. Set $t := \bigsqcup p(x)$. We need to show that $x = t$.

Since *x* is an upper bound and *t* the least upper bound of *p*(*x*), $t \sqsubseteq x$. If $x \sqsubseteq t$, then $x = t$ by anti-symmetry, and we are done. So suppose, for *reductio*, that $x \sqsubseteq t$ is false. Then $t \sqsubset x$, and since $t \ne 0$, Weak Supplementation guarantees that there is *z* with $z \ne 0$ and $z \sqsubseteq x$ that does not overlap *t*. Then not $t \sqsubseteq z$, and since $t \sqsubseteq x$ holds, $z \ne x$. Hence $z \sqsubset x$, which together with $z \ne 0$ gives us $z \in p(x)$. But since *z* and *t* do not overlap, not $z \sqsubseteq t$, so *t* is not an upper bound of *p*(*x*) after all, contradicting $t = \bigsqcup p(x)$. $\square$

In finite frames, the converse also holds:

### **Proposition 6**

*If Proper Fusion holds in a finite mereological frame, so does Weak Supplementation*.

### *Proof*

Define *q*(*x*) as *p*(*x*) if $p(x) \ne \emptyset$, and as $\{x\}$ otherwise. Set $r_0(x) := q(x)$, and $r_{i+1}(x) := \bigcup _{y \in r_i(x)} q(y)$.

Assume that *F* has only finitely many members, and pick *x* and *y* with $y \ne 0$ and $y \sqsubset x$. We show by induction that for every *i*, $r_i(y) \subseteq r_i(x)$. The base step holds because proper parthood is transitive. Suppose that $z \in r_{i+1}(y)$. Then *z* is a member of  *q*(*t*) for some $t \in r_i(y)$. By the induction hypothesis, $t \in r_i(x)$. Hence $z \in r_{i+1}(x)$.

Given Proper Fusion and the associativity of the fusion operation, $x = \bigsqcup r_i(x)$ for every *i*. Since $x \ne y$, $r_i(x) \ne r_i(y)$, and hence $r_i(x) \setminus r_i(y)$ is non-empty for every *i*.

 We can verify that $r_{i+1}(x) \subseteq r_i(x)$, for every *i*. Since *F* has finitely many members, there is *k* such that $r_k(x) = r_{k+1}(x)$. It follows that every member of $r_k(x)$ is atomic, in particular any member *z* of $r_k(x) \setminus r_k(y)$. Then *z* is a part of *x* that does not overlap *y*. Since *x* and *y* were chosen arbitrarily, Weak Supplementation holds. $\square$

Hence Weak Supplementation and Proper Fusion stand and fall together in finite frames.

The converse may fail in the infinite case, however. Consider the (positive and negative) real numbers with $\omega$ and $-\omega$ added, with the usual ordering interpreted as parthood. This is clearly a partial order, and every subset has a least upper bound. Nothing is weakly supplemented in this structure, since any two non-zero elements overlap in their minimum. Nonetheless, every element is the least upper bound of those smaller than it.

## Footnotes

1 For a recent defence of the strategy of using grounding to characterise fundamentality, see Mehta (2017).

2 Wilson uses upper-case “Grounding” for ground, and “grounding relations” (lower case) for the building relations. I take it that that difference is merely notational.

3 The explication matches “the strongest sense of independence” that Bennett contrasts with “notions of independence $\ldots$ that are indexed to a particular building relation”: “A thing (fact, property, what have you) is independent in this strongest sense just in case nothing builds it in any way whatsover; it is not the output of any building relation.” (Bennett 2017, 112)

4 The discussion does not carry over, at least not immediately, to a discussion of the relationship between the ground structure among facts and the fundamentality of entities that are constituents of these facts is in question—the main theme of Raven (2016). For that reason, the frameworks of this article and that of Raven’s cannot be straightforwardly compared.

5 Compare Bennett (2017, 109): “[T]he complete entities are those that build, not *everything*, but everything *else*” (emphasis hers). The notion of a ground base is one conjunct in Kit Fine’s notion of a *distributive ground* for everything (Fine 2012a, p. 54). Moreover, given plausible assumptions, we can show that $\Gamma$ is a grounding base just in case for every *f*, there is $\Gamma ' \subseteq \Gamma$ such that $\Gamma '$ is a *weak ground*, in Fine’s sense, for *f*.

6 Bennett (2017, 123) claims that “independence [B-fundamentality*] seems to me better suited as the central concept of fundamentality.” Her reason is that in a “flat” world, where no facts stand in the relation of ground, we count everything as fundamental because it is ungrounded, despite its failing to ground anything else. This argument seems to me to show only that B-fundamentality is the central distributive, rather than collective, concept of fundamentality. Suppose that we are asked, of all the facts in a flat world, why they are, collectively, the fundamental facts. It then seems to be a good answer to point out that nothing else fails to have a ground among them.

7 Along with many others, both Rosen (2010, p. 116) and Correia (2005, pp. 62–63) endorse this principle, or ones that entail it. See also Kovacs (2018), cf. Jenkins (2011), Bliss (2014).

8 I think that the notion of a building base matches the general notion of completeness that Bennett has in mind: The more intuitive notion of completeness is $\ldots$ one that quantifies over building relations: the complete set at a world is the set whose members build, in one way or another, everything else. (Bennett 2017, 110)

9 There are a variety of “well-foundedness” conditions in the vicinity here, but differences among them do not matter for present purposes (Dixon 2016b; Rabin and Rabern 2016).

10 A rival characterization to my A-fundamentality—one arguably favoured by Bennett, although she is not explicit on the matter—is belonging to some *minimal* grounding base. A detailed comparison between these two proposals would be technical and somewhat orthogonal to the main point of the present paper. I shall merely point out that provided ground satisfies an infinitary version of transitivity (“Cut” in Fine 2012b), then it can be proved that every fact that belongs to some minimal grounding base is A-fundamental. Moreover, facts that are A-fundamental but do not belong to any minimal grounding base are, intuitively, good candidates for being fundamental. In short: the notions will typically coincide, and when they do not, I think A-fundamentality is preferable.

11 See Brzozowski (2008) and Schaffer (2010) in favour of ground always being well-founded; and Schaffer (2003) against.

12 Following Litland (2018), I here use a notion of partial ground that is plural on the left.

13 For relevant discussion concerning ‘because’, see Schnieder (2011, 450).

14 In the views of Wilson (2018a) and Schaffer (2016), there is a very close relationship between ground and causation.

15 For some examples that might be taken to motivate the rejection of that condition (although they are put forward for a different purpose by the author), see Schaffer (2012, 126–9).

16 Here I am indebted to comments by David Liggins.

17 In the words of Williamson (2000, 80): “[L]et $\alpha$ be any case in which a prime condition C obtains, and D and E respectively the strongest narrow condition and the strongest environmental condition which obtain in $\alpha$. Then it is plausible that the conjunction D $\wedge$ E entails C”.

18 A necessary and sufficient condition would be a disjunction of conjunctions of internal and external conditions, which is not in general equivalent to a conjunction of a disjunctive internal condition and a disjunctive external condition.

19 The distinctive kind of explanation in question is metaphysical explanation. Metaphysical explanation does not depend on the conversational context or our epistemic situation. We can thus rule out a contrastive reading of the demand for an explanation of $[G^{+}a]$, along the lines of “why does *a* have positive schmarge rather than negative schmarge?”

20 Armstrong uses the term ‘state of affairs’ where I use ‘fact’.

21 On Armstrong’s theory (2004, 72–73), they may not be: parts of a mereological sum are not in general constituents of that sum. But the very fact that they are not—that the first relatum of the *alling* relation is a mereological sum, not some more structured entity—creates trouble for Armstrong’s theory. He proposes to use *alling* in the analysis of universally quantified statements, as follows:

- “All *A*s are *B*s” is true iff there is something that alls *A* and also alls $A \wedge B$.

But this analysis gives absurd results. Consider “All parts of the universe have a mass of at least 1000 kg,” which is falsified by people, pens, and particles, among others. But it comes out as true under the analysis: there is something, namely the universe, that is the fusion of all parts of the universe, and hence stands in the alling relation to “being a part of the universe”; and that very thing is also the fusion of all parts of the universe that have a mass of at least 1000 kg, and hence stands in the alling relation “being a part of the universe that has a mass of at least 1000 kg”. Things get worse. “All parts of the universe have a mass of less than 1000 kg” also comes out true, under the assumption that the universe has a decomposition into things that each have a mass of less than 1000 kg. (A decomposition into protons, neutrons, electrons, and kindred particles would do the trick.) Together, these two general claims propositions entail the absurd claim that nothing is a part of the universe. The problem would not arise with classes in the place of fusions.

22 Armstrong accepts higher-order states of affairs, but they are supposed to be a “free lunch” given *t*; in our terminology, they are grounded in *t*, rather than being grounds of *t*.

23 Fine eschews appeal to totality facts in favour of appeal to totality claims, but that difference is immaterial here.

24 At least, this is so on the formalization of Whitehead’s theory in Clarke (1981). See Simons (1987, 97–98) for some discussion. Drawing on an unpublished talk of mine, Jessica Wilson (2018b, 2019) mentions such putative failures of weak supplementation in connection with partial grounds that are not parts of full grounds.

25 See Varzi (2016) for references.

26 Cameron uses Humeanism to argue against totality states of affairs, as just noted, and he argues further that a plurality without a totality state of affairs would not be base in the relevant sense. Schaffer’s argument from Humeanism to monism has too many moving parts to be summarised here; for a useful discussion, see Raven (2016).

27 For a detailed discussion of alternative ways of motivating something like Humeanism, see Wang (2016).
